# Improvement cues of lesion absorption using the adjuvant therapy of traditional Chinese medicine Qinbudan tablet for retreatment pulmonary tuberculosis with standard anti-tuberculosis regimen

**DOI:** 10.1186/s40249-020-00660-z

**Published:** 2020-05-07

**Authors:** Shao-Yan Zhang, Ji-You Fu, Xiao-Yan Guo, Ding-Zhong Wu, Tong Zhang, Cui Li, Lei Qiu, Chang-Rong Shao, He-Ping Xiao, Nai-Hui Chu, Qun-Yi Deng, Xia Zhang, Xiao-Feng Yan, Zhao-Long Wang, Zhi-Jie Zhang, Xin Jiang, Yue-Juan Zheng, Pei-Yong Zheng, Hui-Yong Zhang, Zhen-Hui Lu

**Affiliations:** 1 Longhua Hospital Shanghai University of Traditional Chinese Medicine, 725 South Wanping Road, Shanghai, 200032 People’s Republic of China; 2 Shanghai University of Traditional Chinese Medicine, 1200 Cai Lun Road, Shanghai, 201203 People’s Republic of China; 3 School of Pharmacy, Shanghai University of Traditional Chinese Medicine, Shanghai, 201203 People’s Republic of China; 4 Department of Tuberculosis, Shanghai Pulmonary Hospital, Tongji University School of Medicine, Shanghai, 200433 People’s Republic of China; 5 Department of Tuberculosis, Beijing Chest Hospital, Capital Medical University, Beijing, 101149 People’s Republic of China; 6 Department of Tuberculosis, Shenzhen Third People’s Hospital, Shenzhen University School of Medicine, Shenzhen, 518000 People’s Republic of China; 7 Department of Tuberculosis, The Second Hospital of Nanjing, Nanjing, 210003 People’s Republic of China; 8 Department of Tuberculosis, Chongqing Public Health Medical Center, Chongqing, 400036 People’s Republic of China; 9 Jinghua Pharmaceutical Group Co., Ltd, Nantong, 226005 People’s Republic of China; 10 Department of Biostatistics and Department of Epidemiology, School of Public Health, Fudan University, Shanghai, 200433 People’s Republic of China; 11 Department of Immunology and Microbiology, School of Basic Medical Sciences, Shanghai University of Traditional Chinese Medicine, Shanghai, 201203 People’s Republic of China

**Keywords:** Retreatment pulmonary tuberculosis, Traditional Chinese medicine, Randomized-controlled, Trial

## Abstract

**Background:**

China is the second highest pulmonary tuberculosis (PTB) burden country worldwide. However, retreatment of PTB has often developed resistance to at least one of the four first-line anti-TB drugs. The cure rate (approximately 50.0–73.3%) and management of retreatment of PTB in China needs to be improved. Qinbudan decoction has been widely used to treat PTB in China since the 1960s. Previously clinical studies have shown that the Qinbudan tablet (QBDT) promoted sputum-culture negative conversion and lesion absorption. However, powerful evidence from a randomized controlled clinical trial is lacking. Therefore, the aim of this study was to compare the efficacy and safety of QBDT as an adjunct therapy for retreatment of PTB.

**Methods:**

We conducted a multicenter, randomized, double-blind, placebo-controlled clinical trial in China. People diagnosed with PTB were enrolled who received previous anti-TB treatment from April 2011 to March 2013. The treatment group received an anti-TB regimen and QBDT, and the control group was administered an anti-TB regimen plus placebo. Anti-TB treatment options included isoniazid, rifampicin, pyrazinamide, ethambutol, streptomycin for 2 months (2HRZES), followed by isoniazid, rifampicin, ethambutol for 6 months (6HRE), daily for 8 months. Primary outcome was sputum-culture conversion using the MGIT 960 liquid medium method. Secondary outcomes included lung lesion absorption and cavity closure. Adverse events and reactions were observed after treatment. A structured questionnaire was used to record demographic information and clinical symptoms of all subjects. Data analysis was performed by SPSS 25.0 software in the full analysis set (FAS) population.

**Results:**

One hundred eighty-one cases of retreatment PTB were randomly divided into two groups: the placebo group (88 cases) and the QBDT group (93 cases). A total of 166 patients completed the trial and 15 patients lost to follow-up. The culture conversion rate of the QBDT group and placebo group did not show a noticeable improvement by using the covariate sites to correct the rate differences (79.6% vs 69.3%; rate difference = 0.10, 95% confidence interval (*CI)*: - 0.02–0.23; *F* = 2.48, *P* = 0.12) after treatment. A significant 16.6% increase in lesion absorption was observed in the QBDT group when compared with the placebo group (67.7% vs 51.1%; rate difference = 0.17, 95% *CI*: 0.02–0.31; *χ*^2^ = 5.56, *P* = 0.02). The intervention and placebo group did not differ in terms of cavity closure (25.5% vs 21.1%; rate difference = 0.04, 95% *CI*: - 0.21–0.12; *χ*^2^ = 0.27, *P* = 0.60). Two patients who received chemotherapy and combined QBDT reported pruritus/nausea and vomiting.

**Conclusions:**

No significant improvement in culture conversion was observed for retreatment PTB with traditional Chinese medicine plus standard anti-TB regimen. However, QBDT as an adjunct therapy significantly promoted lesion absorption, thereby reducing lung injury due to *Mycobacterium tuberculosis* infection.

**Trial registration:**

This trial is registered at ClinicalTrials.gov, NCT02313610.

## Background

Tuberculosis (TB) is a chronic infectious disease caused by *Mycobacterium tuberculosis* with an estimated incidence of 10 million patients. According to the World Health Organization (WHO), an estimated 1.2 million people died in 2018 due to TB. China is one of the 30 countries with a high-burden of TB (accounting for 9% of all global cases), which occupies the top two slots in terms of death and incidence rate [1]. TB is an important public health issue in China; therefore, major projects of National Science and Technology were dedicated to TB control programs and drug research to improve the rate of cure and reduce the rate of morbidity and mortality.

Retreatment of pulmonary TB (PTB) patients who previously received treatment of at least 1 month with anti-TB drugs involved management of diverse entities, such as relapse, failure, treatment after default, and poor patient adherence to previous treatments [2]. An updated meta-analysis demonstrated that multi-drug resistance among new and retreatment cases was 4.8 and 26.3%, respectively [3]. In a national survey of drug-resistant TB (DRTB) conducted in China, 34.2% of patients developed new TB infections, while 54.5% of previously-treated patients developed resistance to at least one of the four first-line anti-TB drugs [4]. In China, the cure rate for retreatment PTB was approximately 50.0–73.3% [5–7]. In many cases, retreatment represented the patient’s last chance of a cure. The standard anti-TB treatment regimen for retreatment PTB was isoniazid, rifampicin, pyrazinamide, ethambutol, streptomycin for 2 months (2HRZES), followed by isoniazid, rifampicin, ethambutol for 6 months (6HRE), daily for 8 months. It increased the use of second-line injection of streptomycin and extended the consolidation period when compared with newly-treated TB. Several adverse reactions, including gastrointestinal symptoms, liver function impairment, and renal insufficiency often affected the patient’s compliance, which was not conducive to treatment outcome [8].

In China, traditional Chinese medicine (TCM) has been used to treat PTB for thousands of years. Presently, TCM compounds are widely used to treat PTB as a complementary cure in current chemotherapy regimens in China [9]. According to previous studies, it was shown that TCM compounds can increase the absorption of lesions and culture conversion in patients with retreatment PTB [10–12]. Simultaneously, TCM compounds increased immunity and relieved symptoms, especially reducing adverse effects of patients with PTB who were undergoing long-term chemotherapy [13].

In China, QBDT research started in the 1960s when China counted ten million of PTB patients [14]. At the time, first-line drugs were used to treat PTB, however, drug resistance arose rapidly. By consulting ancient texts and clinician’s judgment of more than 1000 cases of PTB from the mid-1960s to the 1980s, the compound decoction of Huangqin (*Scutellaria baicalensis*), Baibu (*Stemona stemonae*), and Danshen (*Salvia miltiorrhizae*) was found to be effective in treating PTB [14, 15]. Based on herbal medicine records, Baibu, Huangqin, and Danshen were important drugs for the treatment of PTB, which improved the cure rate of PTB. To improve drug efficacy and administration, tablets were prepared following an aqueous saturated lime precipitation and ethanol extraction method. The formulation was changed to tablets to improve clinical convenience. QBDT have been widely used in Shanghai since the mid-1980s, even in patients with DRTB [16].

Pharmacological studies on QBDT revealed the active ingredients to be baicalin, tuberostemonine, and tanshinone. Baicalin was identified as a potential inhibitor of the *Mycobacterium tuberculosis* proteasome with more than 65% inhibitory activity [17]. Tuberostemonine has been widely used as an antibacterial drug, especially in complex formulations [18], and may play a role in inhibiting TB resistance by altering the streptomycin resistance protein RpsL (encoding the ribosomal protein S12), and by enhancing the streptomycin-sensitizing effect [19]. Tanshinone is beneficial in the recovery of patients after TB infection by decreasing hypoxic injury and inhibiting calcium accumulation [20]. Both in vitro and in vivo, QBDT may have bacteriostatic effects on TB bacteria by improving hemorheology, inhibiting inflammatory cell infiltration, and toxin secretion [21–23]. According to recent studies, QBDT can upregulate the expression of Toll-like receptor 2, which is one of the mechanisms through which QBDT acts against TB infection [24].

QBDT has bacteriostatic effects on *Mycobacterium tuberculosis* and immune regulatory effects on TB patients, however, its application is limited due to lack of evidence from randomized controlled clinical trials. We hypothesized that current anti-TB treatment regimens for the retreatment of PTB are not potent enough and that outcomes may be improved by increasing culture conversion, lesion absorption, or cavity closure by using additional drugs, such as TCM. Therefore, in this study, a multicenter, double-blind, randomized controlled clinical trial was designed to compare the efficacy and safety of QBDT as an adjunct therapy for the retreatment of PTB.

## Methods

### Study design

To evaluate the efficacy and safety of QBDT as an adjunct therapy for the retreatment of PTB, a multicenter, randomized, double-blind, placebo-controlled trial was conducted in China from April 2011 to March 2013. The trial was designed to enroll patients who were diagnosed with PTB and previously received anti-TB treatment. The primary outcome measurement included the conversion of sputum during the 8-month treatment period, and the secondary outcome measurement included lesion absorption and cavity change.

Twelve sites sentinel hospitals from eleven regions throughout China were recruited and were as follows: Shanghai Pulmonary Hospital affiliated Tongji University, the 85th Hospital of Chinese People’s Liberation Army, Beijing chest hospital affiliated Capital Medical University, the First Affiliated Hospital Of Chongqing Medical University, Shenzhen Donghu Hospital, Hebei Provincial Chest Hospital, Jiangxi Provincial Chest Hospital, Tianjin Haihe Hospital, Shenyang Chest Hospital, Wuhan Tuberculosis Dispensary, the First Affiliated Hospital of Xinxiang Medical University, and the Uygur Autonomous Region of Xinjiang Chest Hospital. The course of the study consisted of two periods: a 1-month screening period and an 8-month primary treatment period. The institutional review board of the Longhua Hospital Shanghai University of TCM formulated and approved the protocol prior to the start of the study.

The estimated sample size was calculated based on the following equation [25]:

$$ \mathrm{n}B=\left(\frac{pA\left(1- pA\right)}{\kappa }+ pB\left(1- pB\right)\right.{\left(\frac{z_{1-\alpha }+{z}_{1-\beta }}{pA- pB}\right)}^2 $$

i. The sputum-culture conversion rate in a standard anti-TB regimen with placebo therapy, _*P*_*A* = 70%
ii. The sputum-culture conversion rate in a standard anti-TB regimen with adjunct QBDT therapy, _*P*_*B* = 90%
iii. Ratio of sample size, k = 1 (1:1)
iv. α = 5%
v. Power of the study (1 − β, % of chance of detecting) = 90%

Finally, n*A* = n*B* = 65, considering the long-term regimen and 35% lost to follow-up, n*A* = n*B* = 65 (1 + 35%) = 88. A total of 176 patients were finally enrolled and signed written informed consent forms.

### Patients’ enrollment

Patients aged between 18 and 65 years with initially culture-positive PTB who had been previously treated were eligible for enrollment. Inclusion criteria involved the following: willingness to be treated, adherence to measurement regimens, and no involvement in another clinical trial 1 month prior and written informed consent. Exclusion criteria included the following: bacterial culture identified as nontuberculous mycobacteria, patients diagnosed with isoniazid-resistant or rifampin-resistant or multidrug-resistant TB, patients who received less than 30 days of treatment, patients with severe primary diseases, patients with mental illness, patients with abnormal liver function, patients with diabetes with poorly controlled plasma glucose levels, female patients in lactation period, during pregnancy, or patients who were planning to get pregnant during the study.

### Drug administration

QBDT and the placebo were prepared by the Jinghua Pharmaceutical Group Co., Ltd. (Lot: 101103) (Nantong, China) and distributed to the 12 sites from board institutional. The same appearance, shape, size, and packaging were used for both test and control drugs. DAS2.0 software (Drug and Statistics, Wannan Medical College, Wuhu, China) was used to generate random numbers with a block size of 4, and all sites could apply for random numbers through the random application DAS central electronic system (DAS for interactive web response system).

The main active ingredients of QBDT (0.35 g) were baicalin, tuberostemonine, and tanshinone. QBDT was processed from 3 g of the original herb, which contained 25% *Scutellaria baicalensis* (0.75 g), 50% *Radix stemonae* (1.5 g), and 25% *Salvia miltiorrhizae* (0.75 g). The dose included four tablets, three times daily, with a total daily dosage of 36 g of the original herbs. The criteria for the quality of the herbs were in accordance with the 2005 Chinese pharmacopoeia [26].

Patients received either QBDT or placebo plus a standard anti-TB treatment on study entry according to the guidelines of the China TB control program [27]. This included treatment for a 2-month intensive phase and 6-month continuation phase with 2HRZES/6HRE (Table 1). If patients were resistant to pyrazinamide or ethambutol, drugs could be adjusted to p-aminosalicylic acid or protionamide. Streptomycin could be replaced with amikacin. Patients who developed resistance to isoniazid and rifampicin were unblinded and excluded from the study.

**Table 1:** Tuberculosis drugs and their application

Drugs	Dosage form	Frequency	Cure approach per day	
			Body weight < 50 kg	Body weight ≥ 50 kg
Isoniazid (H)	Tablet	Once a day	0.3 g	0.3 g
Rifampicin (R)	Capsule	Once a day	0.45 g	0.6 g
Ethambutol (E)	Tablet	Once a day	0.75 g	1.0 g
Pyrazinamide (Z)	Tablet	Once a day	1.5 g	2.0 g
Streptomycin (S)	Injection	Once a day	0.75 g	0.75 g

The double-blind method was applied in doctors and participants. A statistician labeled and distributed drugs to each site and the study coordinator was blinded and assigned participants to treatment using the DAS 2.0 statistical package to generate random codes according to a 1:1 ratio using the central randomization method. Pharmacists at the pharmacy of all clinical trial sites were blinded to the participants’ characteristics and distributed drugs to the patients according to the doctor’s instructions. The selected block length and random initial value seed parameters were sealed as confidential data.

### Outcome measurement

One standardized case report form was designed to obtain sociodemographic characteristics, laboratory and imaging examination data about each retreatment PTB patient. All case report form investigators were trained at a conference to ensure that each site performed the study according to the standard operating procedures. At every visit, spot sputum samples were collected from baseline to 8 months for culture and smear testing. If necessary, induced sputum was collected, where patients were asked to rinse with water, then an ultrasonic atomizer was used to spray 7 ml of 3% hypertonic saline over 15 min and patients attempted to cough up deep sputum. Sputum specimens were digested and decontaminated using the N-acetyl-L-cysteine-sodium hydroxide method. At each clinical laboratory, drug-susceptibility testing was performed of 12 sites by using liquid medium of drug sensitive system of culture and identification of *Mycobacterium* (Becton, Dickinson and Co., NJ, USA) to determine a baseline for all patients [28]. The final concentration of each drug in the culture medium refers to the *Mycobacterium* growth indicator tube operating procedure guide provided by Becton, Dickinson and Company. Each patient underwent chest computed tomography (CT) at the start and end of treatment to evaluate lesion absorption and cavity closure. Safety indicators were analyzed by a monthly review of blood, urine, liver, and kidney function, electrocardiogram and detailed records of drug delivery and timely recording and handling of adverse drug reactions. Fully informed trial content, and free medications and examinations mentioned for patients were conducted to confirm patients’ adherence.

The primary outcome measure was the conversion of sputum between the two groups during the 8-month treatment period for retreatment patients. For sputum-culture conversion [27], the sputum smear and culture needed to be negative during 6, 7, and 8 months of treatment, without any positive results during these 3 months. Secondary outcome measures included lesion absorption and cavity change. The clinical criteria for CT changes were as follows [29]: absorption of 1/2 or more of the lesions was classified as significant absorption, while absorption of less than 1/2 of the lesions or an increase or emergence of new lesions was classified as deterioration. For changes in cavity size, scar healing and block healing or disappearance were defined as closed, while a reduction in cavity size by 1/2 or more was defined as reduced, a reduction in cavity diameter by less than 1/2 was classified as no change, and an increase in the cavity diameter by 1/2 or more was defined as increased. CT results were read and interpreted by two experienced radiologists who were blinded to the study design. If inconsistent, a third expert was invited to re-read. Safety analysis was observed after treatment.

### Data analysis

Full analysis set (FAS) refers to the set of eligible cases and dropout cases, but not excluded cases. When the effect value was missing, it was carried forward with the previous result. Analysis of the primary outcome was TB sputum culture conversion rate. Data for patients that discontinued treatment or did not have sputum-culture conversion were carried forward according to the WHO [30], while patients who dropped out or did not have two consecutive sputum-culture conversion samples during 6–8 months of treatment were considered to have had no response. In addition to the change from baseline to 8-month, pulmonary lesion absorption and cavity closure were assessed as baseline for patients who did not receive a CT test in the 8th month.

The variables of primary and secondary outcome measures were represented by rate differences and 95% confidence intervals *(CI*s). For statistical analysis of the primary endpoint, covariance analysis was employed. The variable “sites” was used as a covariate to correct the rate differences and 95% *CI*s of the sputum-culture conversion. The secondary outcome measures and subgroup analysis were calculated using two tailed Chi square or Fisher’s exact tests.

Safety analysis was performed for adverse events. All participants who had taken at least one dose of the study drugs were included in the safety analysis set. Quantitative variable, including baseline characteristics were described as mean, median, standard deviation or inter-quartile range (IQR), among which the median or mean of quantitative variable was compared by rank-sum test, analysis of variance or *t* test. Data analysis was performed by IBM SPSS software (version 25.0 for Windows, Armonk, NY, USA). All statistical tests were performed using two-tailed test, with *P* < 0.05 considered significant difference.

## Results

### Basic information and clinical symptoms of subjects

**Fig. 1:**
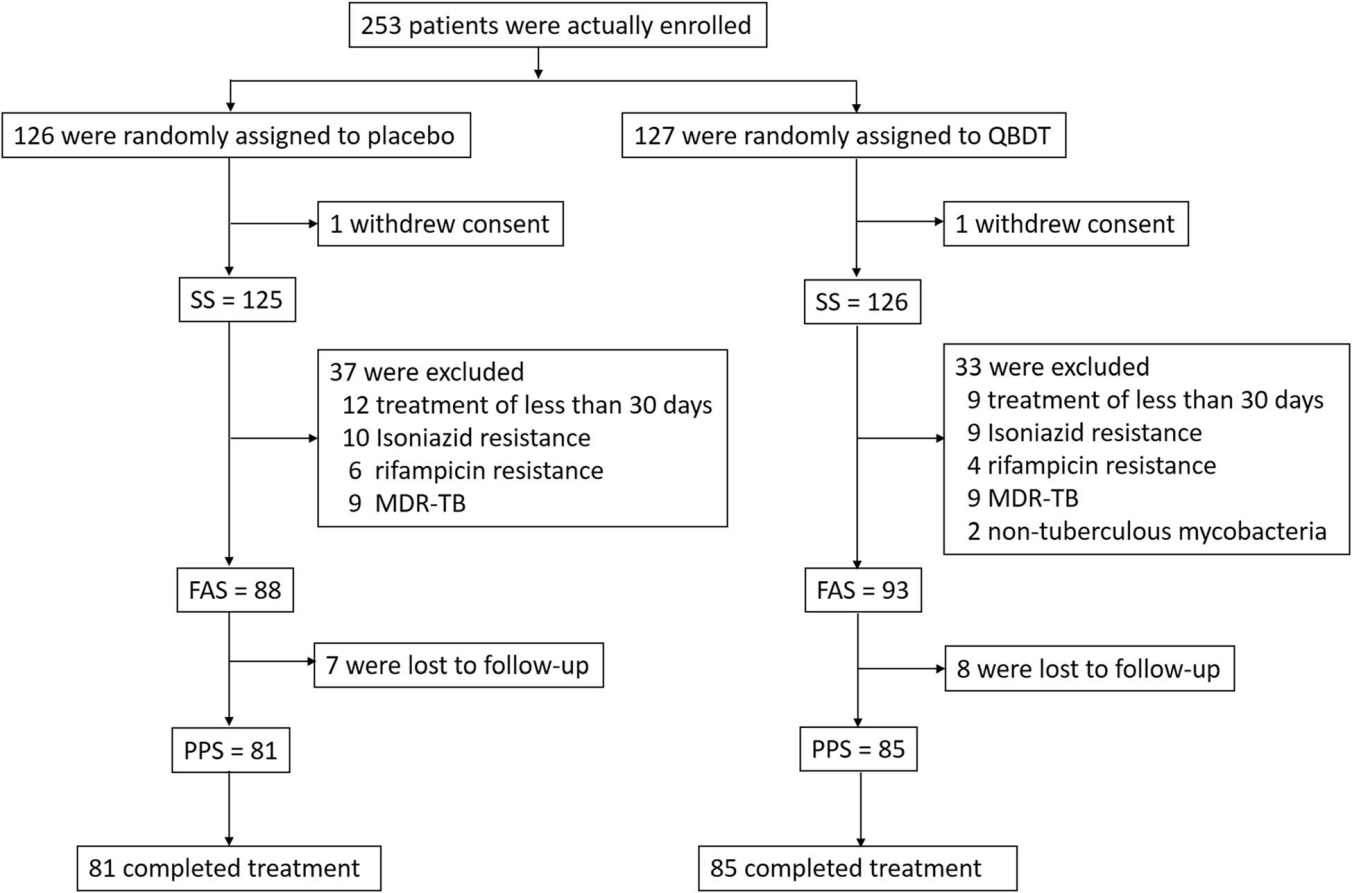
Study profile of enrollment and outcomes. Note: Full analysis set (FAS): refers to a set of qualified cases and loss of cases, but does not include excluded cases. Per protocol set (PPS): refers to patients who meet the inclusion criteria, do not meet the exclusion criteria, and who completed their treatment program. Safety Data Set (SS): patients who received at least one treatment and from whom safety data were recorded. MDR-TB: Multidrug-resistant tuberculosis; QBDT: Qinbudan tablet

Of the 253 patients who were screened, 181 cases of retreatment PTB were eligible and randomly divided into a placebo group (88 cases) and QBDT group (93 cases). The placebo group included 57 males (64.8%) with an average age of 39.30 years and a clinical course of 35.18 months. The QBDT group included 60 males (64.5%) with an average age of 37.72 years and a clinical course of 37.72 months. Most patients in both the placebo and QBDT groups were inpatients (67.0% vs 61.3%), and more than half of them were unemployed, farmers, and workers (53.4% vs 55.9%). Accordingly, the education level ranked first among junior high school students (35.2% vs 35.5%). All patients had a positive *Mycobacterium tuberculosis* culture, and the resistance rate of anti-TB drugs included pyrazinamide, ethambutol, and streptomycin was 17.0, 19.3, 13.6% in the placebo group, and 23.7, 16.1, and 11.8% in the QBDT group respectively. The chest CT examination was abnormal, and more than half of the patients had lung cavitation in the placebo and QBDT groups (59.1% vs 54.8%). Baseline demographic and disease characteristics were similar in the two groups **(**Table 2). A total of 166 patients completed the trial and 15 patients lost to follow-up. In the population, 87 patients (34.4%, 87/253, 95% *CI*: 31.1–43.4) discontinued the trial prematurely, with no relevant differences between the two study groups in the reasons for discontinuation. The most common reasons for treatment discontinuation were poor compliance, exclusion, lost to follow-up, and adverse events (Fig. 1).

**Table 2:** Baseline characteristics of the study population

Characteristics		Placebo *n* = 88 (%)	QBDT *n* = 93 (%)	Statistics^a/b^	*P* value
Sociodemographic characteristics					
Gender^a^	Male	57 (64.8)	60 (64.5)	0.00	0.97
	Female	31 (35.2)	33 (35.5)		
Source of patients	Outpatient	29 (33.0)	36 (38.7)	0.65	0.42
	Inpatient	59 (67.0)	57 (61.3)		
Marital status	Unmarried	30 (34.1)	34 (36.6)	0.12	0.73
	Married	58 (65.9)	59 (63.4)		
Occupation	Worker	13 (14.8)	16 (17.2)	4.84	0.85
	Farmer	15 (17.0)	13 (14.0)		
	Cadre	3 (3.4)	4 (4.3)		
	Intellectual	1 (1.1)	3 (3.2)		
	Soldier	2 (2.3)	3 (3.2)		
	Staff member	10 (11.4)	11 (11.8)		
	Self-employed	5 (5.7)	7 (7.5)		
	Retired	11 (12.5)	5 (5.4)		
	Unemployed	19 (21.6)	23 (24.7)		
	Other	9 (10.2)	8 (8.6)		
Education level	Primary school	16 (18.2)	14 (15.1)	1.20	0.75
	Junior high school	31 (35.2)	33 (35.5)		
	Senior middle School	20 (22.7)	27 (29.0)		
	University and more	21 (23.9)	19 (20.4)		
Age^b^	Mean ± SD	39.30 ± 14.95	37.72 ± 15.33	0.70	0.49
Height (cm)	Mean ± SD	166.96 ± 6.56	167.83 ± 8.80	0.75	0.45
Weight (kg)	Mean ± SD	56.05 ± 10.06	55.77 ± 10.08	0.19	0.85
Complications	No	68 (77.3)	77 (82.8)	0.87	0.35
	Yes	20 (22.7)	16 (17.2)		
Clinical course	Mean ± SD	35.18 ± 47.02	37.72 ± 46.30	0.37	0.72
Laboratory and imaging examination					
Sputum smear and culture	Culture+ smear+	74 (84.1%)	81 (87.1%)	0.33	0.56
	Culture+ smear-	14 (15.9%)	12 (12.9%)		
Drug resistance	Pyrazinamide	15 (17.0%)	22 (23.7%)	1.22	0.27
	Ethambutol	17 (19.3%)	15 (16.1%)	0.32	0.57
	Streptomycin	12 (13.6%)	11 (11.8%)	0.13	0.72
Lung lesion	Normal	0 (0.0)	0 (0.0)	0	1.00
	Abnormal	88 (100.0)	93 (100.0)		
Cavitation	No	36 (40.9)	42 (45.2)	0.33	0.56
	Yes	52 (59.1)	51 (54.8)		

Note: ^a^chi-square test, statistics value, *χ*^2^; ^b^rank sum test statistics value, Z; *QBDT*: Qinbudan tablet; *CT*: Computed tomography

### Sputum-culture conversion (the primary endpoint)

Comparison of the sputum-culture convention rate in placebo and QBDT groups at each site are shown in Table 3. At 8 months after the end of treatment, the total culture conversion rate of the QBDT group was 79.6% (74/93, 95% *CI*: 70.3–86.5), which was 10.3% higher when compared with the placebo group (69.3%, 61/88, 95% *CI*: 59.1–78.0). However, the differences were not significant (rate difference = 0.10, 95% *CI*: − 0.02–0.23; *F* = 2.48, *P* = 0.12) when using the covariate sites to correct the rate differences. It is well known that the sensitivity of anti-TB drugs is crucial for the negative conversion of sputum, therefore, subgroup analysis was performed based on the sensitivity of the drugs. As shown in Table 4, the adjuvant therapy of QBDT improved the sputum negative conversion in patients who were sensitive or resistant to pyrazinamide, ethambutol, and streptomycin. None of the patients showed significant negative conversion, although patients who were sensitive to pyrazinamide tended to significantly improve the negative conversion.

**Table 3:** Comparison of the sputum-culture convention rate in placebo and QBDT group in each site

Sites	Placebo (*n* = 88)				QBDT (*n* = 93)			
	Negative culture *n*	Positive culture *n*	Withdrawal *n*	Negative rate	Negative culture *n*	Positive culture *n*	Withdrawal *n*	Negative rate
1	1	3	2	16.7%	0	1	0	0.0%
2	1	0	0	100.0%	1	0	0	100.0%
3	0	0	0	0.0%	1	1	0	50.0%
4	3	0	0	100.0%	6	1	2	66.7%
5	3	7	2	25.0%	9	3	1	69.2%
6	5	1	0	83.3%	4	0	1	80.0%
7	7	1	0	87.5%	8	0	0	100.0%
8	4	3	1	50.0%	2	1	3	33.3%
9	9	0	1	90.0%	11	0	0	100.0%
10	15	3	0	83.3%	17	0	2	89.5%
11	11	1	0	91.7%	13	0	1	92.9%
12	2	1	1	50.0%	2	1	1	50.0%
Total	61	20	7	69.3%	74	8	11	79.6%

*QBDT* Qinbudan tablet; Covariance analysis was used to correct the rate differences of primary endpoints. The covariate variable sites as follows: 1 Shanghai pulmonary hospital affiliated Tongji University, 2 Beijing chest hospital affiliated Capital medical university, 3 the first affiliated hospital of Chongqing medical university, 4 Shenzhen Donghu hospital, 5 Hebei provincial chest hospital, 6 Jiangxi provincial chest hospital, 7 Tianjin Haihe hospital, 8 Shenyang chest hospital, 9 Wuhan tuberculosis dispensaries, 10 the first affiliated hospital of Xinxiang Medical University, 11 Uygur Autonomous Region of Xinjiang Chest Hospital, 12 the 85th hospital of Chinese people’s liberation army

**Table 4:** Comparison of the sputum-culture convention rate in placebo and QBDT group in subgroup by drug sensitivity

		Placebo (*n* = 88)			QBDT (*n* = 93)			*OR* (95% *CI*)	*P* value
Drug sensitivity		Negative *n* (%)	Positive *n* (%)	Withdrawal *n* (%)	Negative *n* (%)	Positive *n* (%)	Withdrawal *n* (%)		
Pyrazinamide	Sensitive	56 (76.7)	11 (15.1)	6 (8.2)	63 (88.7)	3 (4.2)	5 (7.0)	0.42 (0.17–1.04)	0.06
	Resistant	5(33.3)	9 (60.0)	1 (6.7)	11(50.0)	5 (22.7)	6 (27.3)	0.50 (0.13–1.95)	0.32
Ethambutol	Sensitive	52 (73.2)	16 (22.5)	3 (4.2)	64 (82.1)	5 (6.4)	9 (11.5)	0.60 (0.27–1.31)	0.20
	Resistant	9 (52.9)	4 (23.5)	4 (23.5)	10 (66.7)	3 (20.0)	2 (13.3)	0.56 (0.13–2.36)	0.43
Streptomycin	Sensitive	59 (77.6)	12 (15.8)	5 (6.6)	70 (85.4)	4 (4.9)	8 (9.8)	0.60 (0.26–1.35)	0.21
	Resistant	2 (16.7)	8 (66.7)	2 (16.7)	4 (36.4)	4 (36.4)	3 (27.3)	0.35 (0.05–2.47)	0.28
Total		61 (69.3)	20 (22.7)	7 (8.0)	74 (79.6)	8 (8.6)	11 (11.8)	0.58 (0.30–1.14)	0.11

*QBDT* Qinbudan tablet

### Lesion absorption (the secondary endpoint)

Of the 88 patients in the placebo group (significantly absorbed in 45 cases, absorbed in 31 cases, no change in 11 cases, deterioration in 1 case), the significant absorption rate was 51.1% (45/88, 95% *CI*: 40.9–61.3**)**. Moreover, of the 93 patients in the QBDT group (significantly absorbed in 63 cases, absorbed in 25 cases, no change in 5 cases, deterioration in 0 case), the significant absorption rate was 67.7% (63/93, 95% *CI*: 57.7–76.4**)**. A significant increase of 16.6% lesion absorption was observed in the QBDT group when compared with placebo group (rate difference = 0.17, 95% *CI*: 0.02–0.31; *χ*^2^ = 5.56, *P* = 0.02) (Table 5).

**Table 5:** Comparison of significantly lesions absorption in placebo and QBDT group

Group	Lesions absorption				*OR* (95% *CI*)	*P* value
	Significantly absorbed *n* (%)	Absorbed *n* (%)	No change *n* (%)	Deterioration *n* (%)		
Placebo (*n* = 88)	45 (51.1)	31 (35.2)	11 (12.5)	1 (1.1)	0.50 (0.27–0.91)	0.02
QBDT (*n* = 93)	63 (67.7)	25 (26.9)	5 (5.4)	0 (0)		

*QBDT* Qinbudan tablet

### Cavity closure (the secondary endpoint)

In the two groups of patients, there were 103 cases with cavities. In the placebo group (cavity closure in 11 cases, narrow in 1 case, no change in 38 cases, increased in 2 cases), the cavity closure rate was 21.2% (11/52, 95% *CI*: 12.2–34.0). In the QBDT group (cavity closure in 13 cases, narrow in 5 cases, no change in 33 cases, increased in 0 cases) the cavity closure rate was 25.5% (13/51, 95% *CI*: 15.6–38.7). When compared to the placebo group, the closure rate increased by 4% and no significant difference was observed between the groups (rate difference = 0.04, 95% *CI*: − 0.21–0.12; *χ*^2^ = 0.27, *P* = 0.60) (Table 6).

**Table 6:** Comparison of cavity closure in placebo and QBDT group

Group	Cavity closure				*OR* (95% *CI*)	*P* value
	Cavity closure *n* (%)	Narrow *n* (%)	No change *n* (%)	Increased *n* (%)		
Placebo (*n* = 52)	11 (21.2%)	1(1.9%)	38(73.1%)	2(3.8%)	0.78 (0.31–1.96)	0.60
QBDT (*n* = 51)	13(25.5%)	5(9.8%)	33(64.7%)	0(0%)		

*QBDT* Qinbudan tablet

### Safety analysis

In both groups the rates of adverse events and treatment-related adverse reactions were similar. In the placebo group, there were 84 cases (67.2%, 84/125, 95% *CI*: 58.6–74.8) of adverse events, while there were 76 cases (60.3%, 76/126, 95% *CI*: 51.6–68.4) in the treatment group. The adverse reactions mainly included abnormalities in laboratory parameters. In addition, there were three cases (2.4%, 3/125, 95% *CI*: 0.8–5.8) with signs of conjunctival hyperemia/bloating and vomiting/leucopenia in the placebo group, and two cases (1.6%, 2/126, 95% *CI*: 0.4–5.6) including pruritus/nausea and vomiting in the treatment group. No significant differences (*P* > 0.05) in adverse events and adverse reaction rates were observed in the two groups (Additional file 1).

## Discussion

In the registered multicenter, randomized, double-blind, placebo-controlled trial, QBDT as an adjunct therapy significantly promoted lesion absorption. No significant differences between adverse events and adverse reaction rates were observed between the two groups. In addition, no significant differences in the reduction of sputum-culture negative conversion and cavity closure were observed in this trial.

In the present study, the cure rate of chemotherapy for the retreatment of PTB was 69.3%, which was consistent with the data presented by previously reported studies (50.0–73.3%) [5–7]. However, no statistically significant differences were observed with adjunct QBDT therapy. This may be explained as follows. Firstly, the quality control of sputum smear and culture might be imperfect leading to inaccurate results in a multicenter, such as sputum collection and the sputum culture kit used. Secondly, the dosage forms of the tablets and decoction were different in some of the components. Apart from *Scutellaria baicalensis*, *Radix stemonae,* and *Salvia miltiorrhizae*, other ingredients may play an additional role.

In addition, considering the important effect of anti-TB drug sensitivity on sputum negative conversion, subgroup analysis was performed to determine its effect on the two groups. The data demonstrated that adjuvant therapy using QBDT improved the sputum negative conversion in patients who were sensitive or resistant to pyrazinamide, ethambutol, streptomycin, thereby suggesting that QBDT may not directly act on bacteria and primarily activated host immunity to clear a *Mycobacterium tuberculosis* infection. Surprisingly, patients who were sensitive to pyrazinamide tended to significantly improve the negative conversion rate. It is currently recognized that the addition of pyrazinamide to the anti-TB regimen can shorten the long-term treatment course, which is equivalent to the “guide herbs” in the compatibility of TCM prescriptions. Therefore, in pyrazinamide-sensitive patients, the two “guide herbs” including pyrazinamide and QBDT may show a stronger sputum negative culture effect. Although we did not observe a significant difference overall, there was a trend to improvement in sputum conversion.

Furthermore, QBDT was found to significantly promote lesion absorption as a complementary therapy for retreatment PTB. Scar tissue associated with PTB can affect the long-term health of patients, especially those with immunocompromised relapse. Therefore, studies to reduce lung injury due to *Mycobacterium tuberculosis* infection and novel ways to improve the sputum-culture negative conversion should be investigated. For example, TB might be the most important secondary causes of bronchiectasis.

In several studies published in Chinese journals, the efficacy of TCM in adjuvant treatment of retreatment PTB, such as Baihe Gujin decoction [10], Laokang decoction [11] and Yifeizhike capsule [12] was also observed. After combining these TCM formulas, the sputum negative conversion or pulmonary lesion absorption in patients was significantly improved, and was increased by 19.8–20.8% and 12.5–17.6%, respectively. Although the sputum negative conversion had been improved significantly, these studies had some limitations. In short, the evaluation of sputum negative conversion was not based on at least two negative conversions recommended by the WHO in the last 3 months of treatment; the study design did not implement blinded methods, which increased the information bias of the study results; the chemotherapy regimens were not conducted in accordance with the standard anti-TB regimen recommended by WHO, included the use of rifabutin, rifapentin, or the addition of levofloxacin, gatifloxacin, etc.

The mechanism of TCM and its effects on retreatment PTB are complex. According to a study on curcumin enhancing the control of *Mycobacterium tuberculosis* in macrophage, it was shown that TCM may have multi-aspect therapeutic effects on TB, for example, in the aspect of antibacterial activity, and even the regulation of immune inflammation [31]. TCM might mainly focus on the pathological aspects of disease, such as the immune inflammatory response, including systemic and localized lesions caused by *Mycobacterium tuberculosis*, changes in blood circulation, and the body’s metabolic state, in addition to the removal of pathogenic micro-organisms. The beneficial characteristics of TCM allow for more of a “holistic view”, and pay more attention to the overall changes in the body’s disease state. However, this feature will bring the decoction involved several or even dozens of fried herbs mixed, which makes it difficult to use a single target to explain the underlying mechanisms involved.

The present study had several limitations. Firstly, our small sample size did not show a significant difference between the two groups regarding the primary efficacy endpoint. Therefore, we hope that for a phase III study a larger number of patients will be enrolled to further verify the study results. Secondly, to overcome the differences between tablets and decoction in some of the components, we may include a separate QBD decoction group in the upcoming phase III clinical trials design. The test results showed significant differences in lung lesion absorption rates, however, the lesion size as determined by chest CT was relatively subjective, although these were taken into consideration using the Delphi method.

## Conclusions

In conclusion, the addition of QBDT to the retreatment of PTB may result in significant lesion absorption. However, no significant improvement of culture conversion was observed for the retreatment of PTB with QBDT plus standard anti-TB regimen. The significant improvement in lesion absorption indicated the next research direction. Furthermore, we will further optimize the formula at the cell and animal level to improve the advantages of TCM in adjuvant treatment in the retreatment of PTB.

## Supplementary information

**Additional file 1.**

## Data Availability

Data of the study can be available upon request from Zhen-Hui Lu.

## Abbreviations

CT: Computed tomography
*CI*: Confidence interval
DRTB: Drug-resistant tuberculosis
E: Ethambutol
FAS: Full analysis set
H: Isoniazid
IQR: Inter-quartile range
*OR*: Odds ratio
PTB: Pulmonary tuberculosis
QBDT: Qinbudan tablets
R: Rifampicin
S: Streptomycin
TB: Tuberculosis
TCM: Traditional Chinese medicine
Z: Pyrazinamide
