# Supplementary material for: Improvement cues of lesion absorption using the adjuvant therapy of traditional Chinese medicine Qinbudan tablet for retreatment pulmonary tuberculosis with standard anti-tuberculosis regimen
Source: Infect Dis Poverty. 2020 May 7;9:50. doi: 10.1186/s40249-020-00660-z (PMC7203794; doi:10.1186/s40249-020-00660-z)
Supplement: Supplementary file 1 — Additional file 1. [file 40249_2020_660_MOESM1_ESM.docx]

**Supplementary Appendix**

This appendix has been provided by the authors to give readers additional information about their work.

**Appendix 1 safety**

**Appendix 1.1** The Incidence of adverse events and adverse reactions

| Index |  | Placebo  *n* (%) | QBDP  *n* (%) | χ2 | *P* |
| --- | --- | --- | --- | --- | --- |
| Adverse events | Yes | 84(67.2) | 76(60.3) | 0.639 | 0.424 |
|  | No | 41(32.8) | 50(39.7) |  |  |
|  | Total | 125 | 126 |  |  |
| Adverse reactions | Yes | 3(2.4) | 2(1.6) | 0.000 | 1.000 |
|  | No | 122(97.6) | 124(98.4) |  |  |
|  | Total | 125 | 126 |  |  |

**Appendix 1.2** Adverse events in different systems

|  | | Placebo (*n* =125) | | | QBDP (*n* =126) | | | |
| --- | --- | --- | --- | --- | --- | --- | --- | --- |
| System | Adverse events | Cases  *n* | Frequency  *n* | Incidence  (%) | Cases  *n* | Frequency *n* | | Incidence (%) |
| Laboratory tests |  | 79 | 150 | 63.2% | 71 | 145 | 56.3% | |
|  | Elevated erythrocyte | 1 | 1 | 0.8% | 0 | 0 | 0.0% | |
|  | Lower hemoglobin | 0 | 0 | 0.0% | 1 | 1 | 0.8% | |
|  | Elevated hemoglobin | 0 | 0 | 0.0% | 1 | 1 | 0.8% | |
|  | Lower leukocyte | 5 | 5 | 4.0% | 2 | 2 | 1.6% | |
|  | Elevated leukocyte | 2 | 2 | 1.6% | 3 | 3 | 2.4% | |
|  | Elevated platelet | 3 | 5 | 2.4% | 3 | 3 | 2.4% | |
|  | Lower platelet | 1 | 1 | 0.8% | 1 | 1 | 0.8% | |
|  | Elevated uric acid | 31 | 32 | 24.8% | 20 | 21 | 15.9% | |
|  | Elevated urinary red blood cells | 14 | 16 | 11.2% | 26 | 34 | 20.6% | |
|  | Urine leukocytosis | 14 | 15 | 11.2% | 10 | 11 | 7.9% | |
|  | Elevated urinary protein | 11 | 12 | 8.8% | 9 | 11 | 7.1% | |
|  | Elevated urine | 3 | 6 | 2.4% | 2 | 3 | 1.6% | |
|  | Elevated alanine aminotransferase | 8 | 8 | 6.4% | 11 | 12 | 8.7% | |
|  | Elevated aspartate aminotransferase | 9 | 9 | 7.2% | 9 | 10 | 7.1% | |
|  | Lower total bilirubin | 0 | 0 | 0.0% | 2 | 2 | 1.6% | |
|  | Elevated total bilirubin | 5 | 5 | 4.0% | 3 | 3 | 2.4% | |
|  | Lower blood urea nitrogen | 1 | 1 | 0.8% | 1 | 1 | 0.8% | |
|  | Elevated blood urea nitrogen | 1 | 1 | 0.8% | 1 | 1 | 0.8% | |
|  | Lower creatinine | 3 | 3 | 2.4% | 2 | 2 | 1.6% | |
|  | Elevated creatinine | 3 | 3 | 2.4% | 0 | 0 | 0.0% | |
| Respiratory system |  | 6 | 6 | 4.7 | 4 | 5 | 3.2% | |
|  | Upper respiratory tract infection | 3 | 3 | 2.4 | 1 | 1 | 0.8% | |
|  | Spontaneous | 2 | 2 | 1.6 | 2 | 2 | 1.6% | |
|  | Lung infection | 1 | 1 | 0.8 | 0 | 0 | 0.0% | |
|  | Lung inflammation | 0 | 0 | 0.0 | 1 | 2 | 0.8% | |
| Digestive System |  | 5 | 6 | 3.9 | 2 | 4 | 1.6% | |
|  | Vomit | 1 | 1 | 0.8 | 2 | 2 | 1.6% | |
|  | Impaired liver function | 3 | 3 | 2.4 | 0 | 0 | 0.0% | |
|  | Nausea | 0 | 0 | 0.0 | 2 | 2 | 1.6% | |
|  | Bloating | 1 | 1 | 0.8 | 0 | 0 | 0.0% | |
|  | Gastrointestinal discomfort | 1 | 1 | 0.8 | 0 | 0 | 0.0% | |
| Skin System |  | 2 | 2 | 1.6 | 4 | 4 | 3.2% | |
|  | Pruritus | 1 | 1 | 0.8 | 2 | 2 | 1.6% | |
|  | Rash | 1 | 1 | 0.8 | 1 | 1 | 0.8% | |
|  | Ethambutol drug rash | 0 | 0 | 0.0 | 1 | 1 | 0.8% | |
| Urinary System |  | 1 | 1 | 0.8 | 0 | 0 | 0.0% | |
|  | Renal dysfunction | 1 | 1 | 0.8 | 0 | 0 | 0.0% | |
| Other |  | 7 | 7 | 5.5 | 7 | 7 | 5.6% | |
|  | Fever | 1 | 1 | 0.8 | 1 | 1 | 0.8% | |
|  | Allergy | 1 | 1 | 0.8 | 1 | 1 | 0.8% | |
|  | Hypokalemia | 0 | 0 | 0.0 | 1 | 1 | 0.8% | |
|  | Tinnitus | 0 | 0 | 0.0 | 1 | 1 | 0.8% | |
|  | Arthralgia | 1 | 1 | 0.8 | 0 | 0 | 0.0% | |
|  | Eye pain | 1 | 1 | 0.8 | 0 | 0 | 0.0% | |
|  | Double toe joint pain | 1 | 1 | 0.8 | 0 | 0 | 0.0% | |
|  | Dizziness | 0 | 0 | 0.0 | 1 | 1 | 0.8% | |
|  | Personality changes | 0 | 0 | 0.0 | 1 | 1 | 0.8% | |
|  | Chest wall abscess | 0 | 0 | 0.0 | 1 | 1 | 0.8% | |
|  | Conjunctival hyperemia | 1 | 1 | 0.8 | 0 | 0 | 0.0% | |
|  | Drug fever | 1 | 1 | 0.8 | 0 | 0 | 0.0% | |

**Appendix 1.3** Adverse reactions in different systems

|  | | Placebo (*n* =125) | | | QBDP (*n* =126) | | |
| --- | --- | --- | --- | --- | --- | --- | --- |
| System | Adverse reactions | Cases  *n* | Frequency  *n* | Incidence  (%) | Cases  *n* | Frequency  *n* | Incidence  (%) |
| Digestive System |  | 1 | 2 | 0.8% | 1 | 2 | 0.8% |
|  | Vomit | 1 | 1 | 0.8% | 1 | 1 | 0.8% |
|  | Nausea | 0 | 0 | 0.0% | 1 | 1 | 0.8% |
|  | Bloating | 1 | 1 | 0.8% | 0 | 0 | 0.0% |
| Skin System |  | 0 | 0 | 0.0% | 1 | 1 | 0.8% |
|  | Pruritus | 0 | 0 | 0.0% | 1 | 1 | 0.8% |
| Laboratory tests |  | 1 | 1 | 0.8% | 0 | 0 | 0.0% |
|  | Leukopenia | 1 | 1 | 0.8% | 0 | 0 | 0.0% |
| Other |  | 1 | 1 | 0.8% | 0 | 0 | 0.0% |
|  | Conjunctival hyperemia | 1 | 1 | 0.8% | 0 | 0 | 0.0% |
